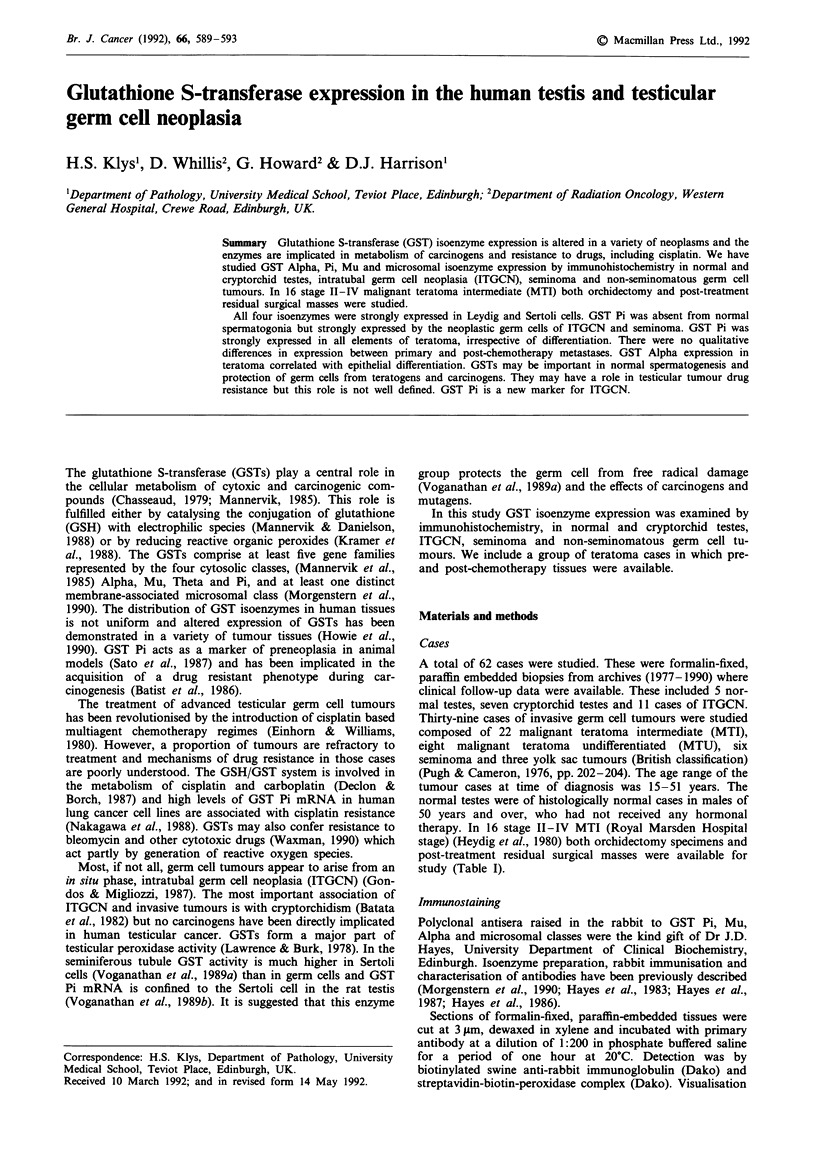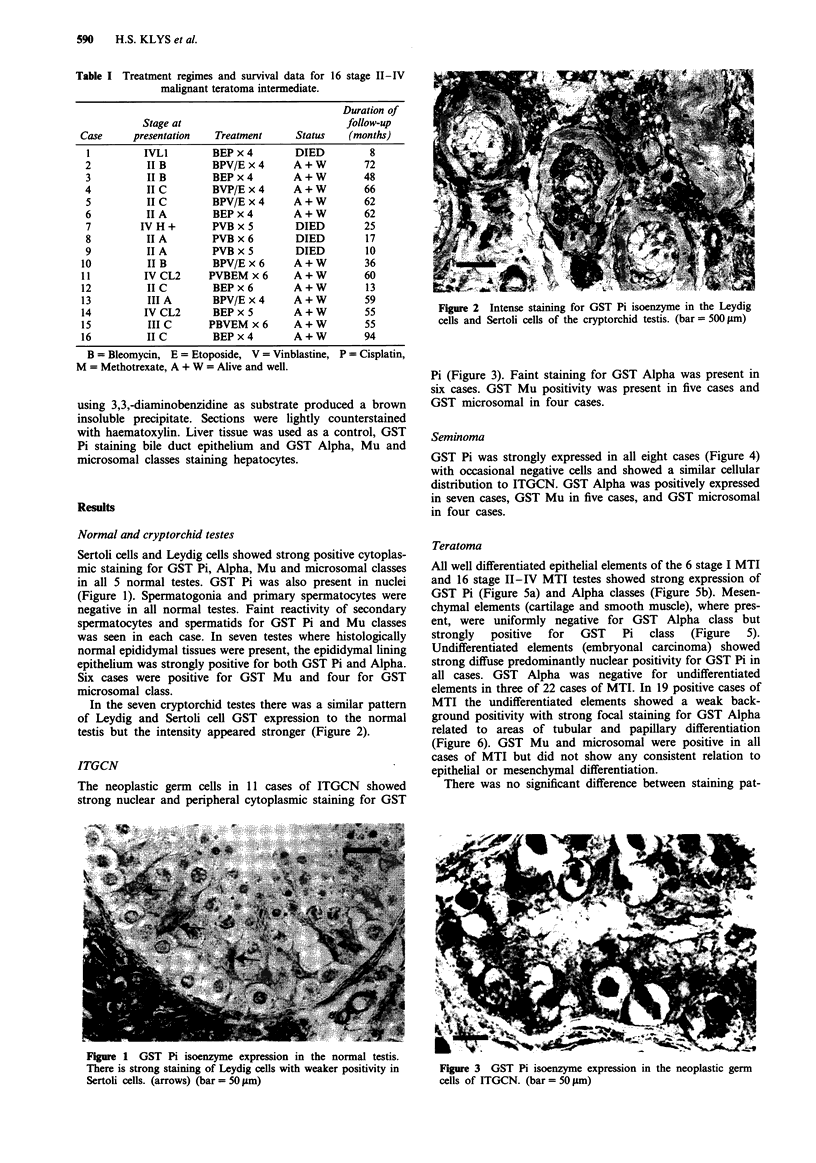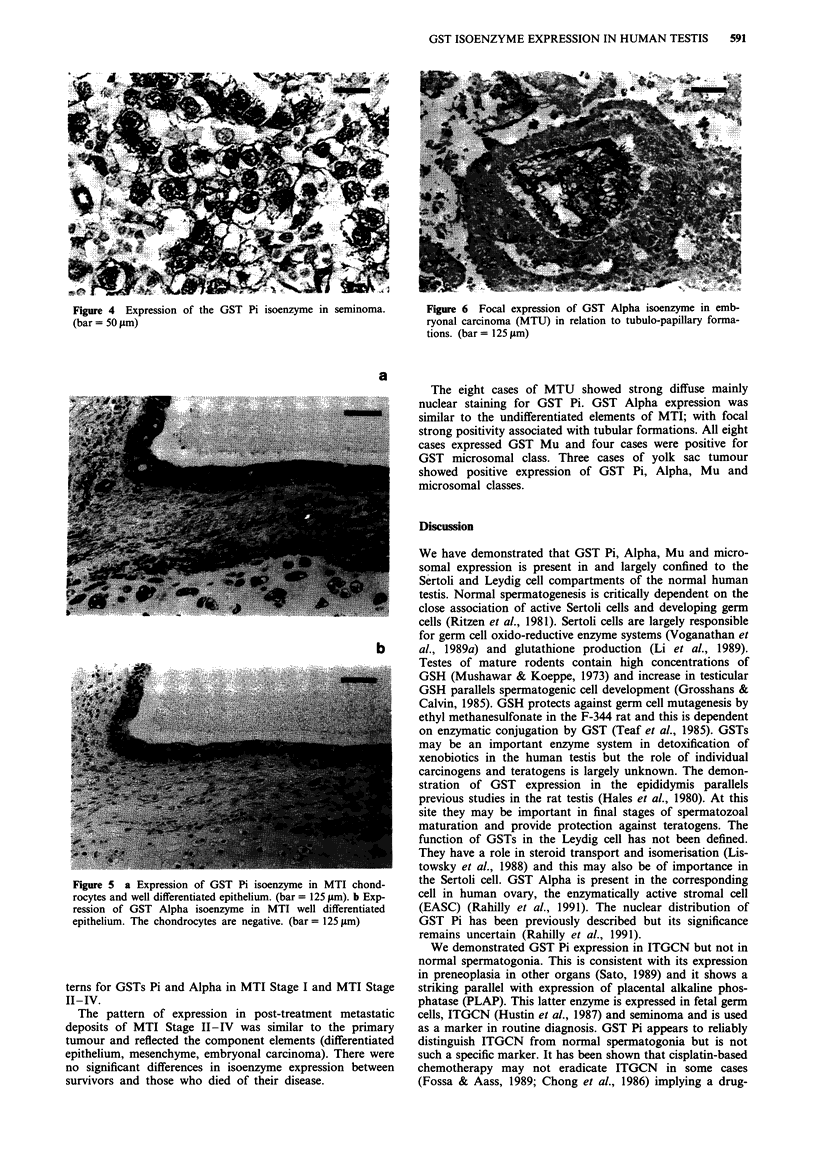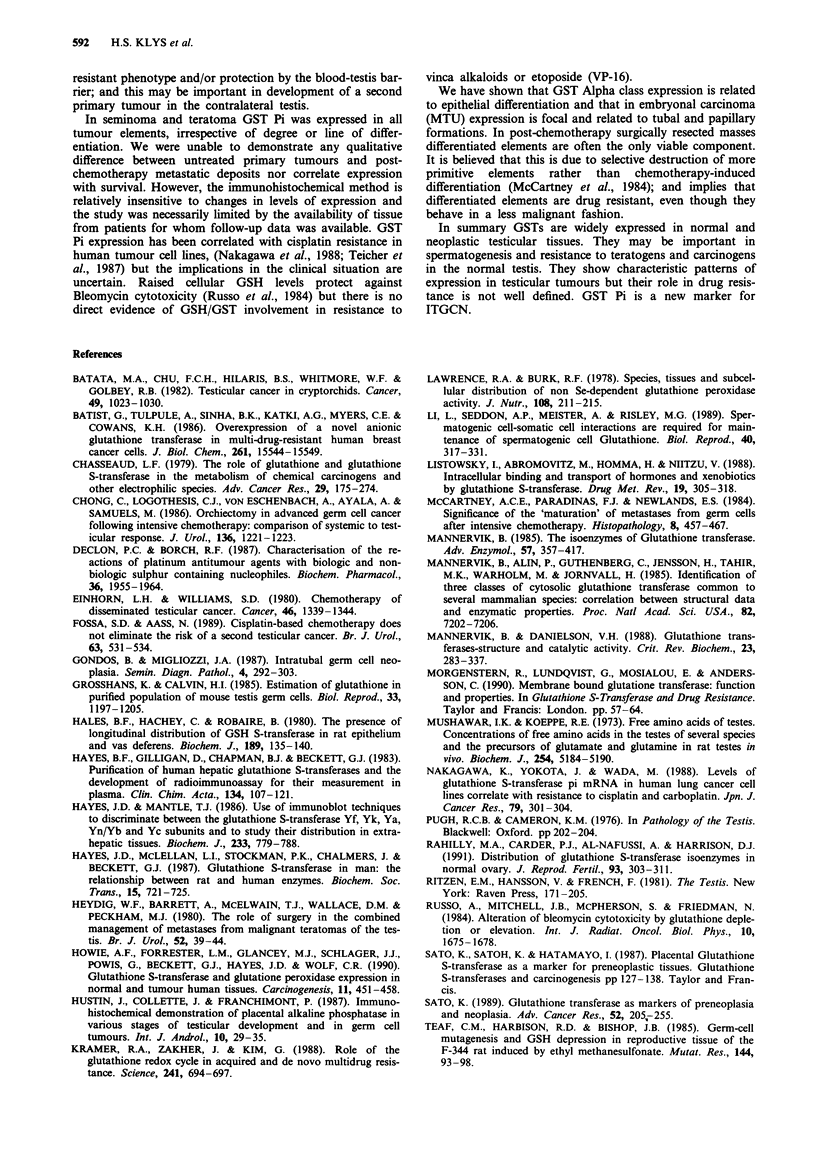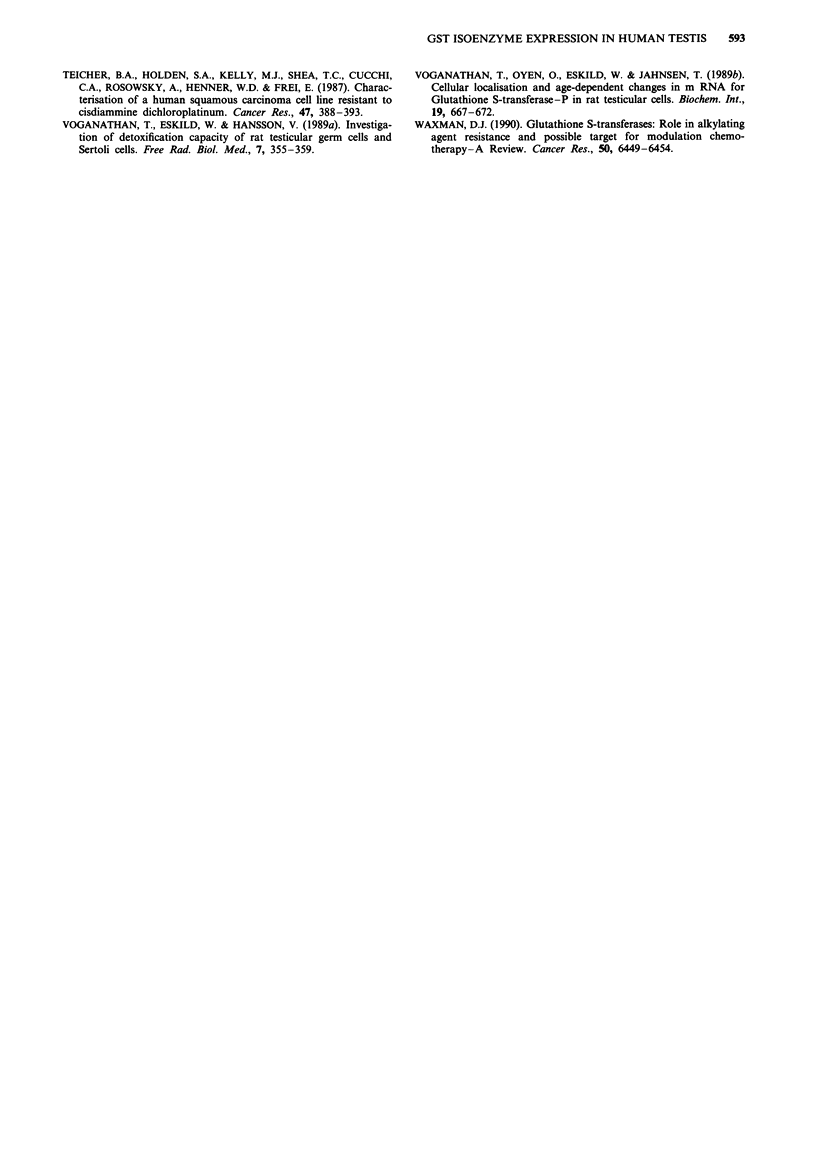# Glutathione S-transferase expression in the human testis and testicular germ cell neoplasia.

**DOI:** 10.1038/bjc.1992.319

**Published:** 1992-09

**Authors:** H. S. Klys, D. Whillis, G. Howard, D. J. Harrison

**Affiliations:** Department of Pathology, University Medical School, Edinburgh, UK.

## Abstract

**Images:**


Br. J. Cancer (1992), 66, 589-593 © Macmillan Press Ltd., 1992

### Glutathione S-transferase expression in the human testis and testicular germ cell neoplasia

H.S. Klys[1], D. Whillis[2], G. Howard[2] & D.J. Harrison[1]

[1]*Department of Pathology, University Medical School, Teviot Place, Edinburgh;* [2]*Department of Radiation Oncology, Western General Hospital, Crewe Road, Edinburgh, UK.*

**Summary** Glutathione S-transferase (GST) isoenzyme expression is altered in a variety of neoplasms and the enzymes are implicated in metabolism of carcinogens and resistance to drugs, including cisplatin. We have studied GST Alpha, Pi, Mu and microsomal isoenzyme expression by immunohistochemistry in normal and cryptorchid testes, intratubal germ cell neoplasia (ITGCN), seminoma and non-seminomatous germ cell tumours. In 16 stage II–IV malignant teratoma intermediate (MTI) both orchidectomy and post-treatment residual surgical masses were studied.

All four isoenzymes were strongly expressed in Leydig and Sertoli cells. GST Pi was absent from normal spermatogonia but strongly expressed by the neoplastic germ cells of ITGCN and seminoma. GST Pi was strongly expressed in all elements of teratoma, irrespective of differentiation. There were no qualitative differences in expression between primary and post-chemotherapy metastases. GST Alpha expression in teratoma correlated with epithelial differentiation. GSTs may be important in normal spermatogenesis and protection of germ cells from teratogens and carcinogens. They may have a role in testicular tumour drug resistance but this role is not well defined. GST Pi is a new marker for ITGCN.

The glutathione S-transferase (GSTs) play a central role in the cellular metabolism of cytoxic and carcinogenic compounds (Chasseaud, 1979; Mannervik, 1985). This role is fulfilled either by catalysing the conjugation of glutathione (GSH) with electrophilic species (Mannervik & Danielson, 1988) or by reducing reactive organic peroxides (Kramer *et al.*, 1988). The GSTs comprise at least five gene families represented by the four cytosolic classes, (Mannervik *et al.*, 1985) Alpha, Mu, Theta and Pi, and at least one distinct membrane-associated microsomal class (Morgenstern *et al.*, 1990). The distribution of GST isoenzymes in human tissues is not uniform and altered expression of GSTs has been demonstrated in a variety of tumour tissues (Howie *et al.*, 1990). GST Pi acts as a marker of preneoplasia in animal models (Sato *et al.*, 1987) and has been implicated in the acquisition of a drug resistant phenotype during carcinogenesis (Batist *et al.*, 1986).

The treatment of advanced testicular germ cell tumours has been revolutionised by the introduction of cisplatin based multiagent chemotherapy regimes (Einhorn & Williams, 1980). However, a proportion of tumours are refractory to treatment and mechanisms of drug resistance in those cases are poorly understood. The GSH/GST system is involved in the metabolism of cisplatin and carboplatin (Declon & Borch, 1987) and high levels of GST Pi mRNA in human lung cancer cell lines are associated with cisplatin resistance (Nakagawa *et al.*, 1988). GSTs may also confer resistance to bleomycin and other cytotoxic drugs (Waxman, 1990) which act partly by generation of reactive oxygen species.

Most, if not all, germ cell tumours appear to arise from an *in situ* phase, intratubal germ cell neoplasia (ITGCN) (Gondos & Migliozzi, 1987). The most important association of ITGCN and invasive tumours is with cryptorchidism (Batata *et al.*, 1982) but no carcinogens have been directly implicated in human testicular cancer. GSTs form a major part of testicular peroxidase activity (Lawrence & Burk, 1978). In the seminiferous tubule GST activity is much higher in Sertoli cells (Voganathan *et al.*, 1989*a*) than in germ cells and GST Pi mRNA is confined to the Sertoli cell in the rat testis (Voganathan *et al.*, 1989*b*). It is suggested that this enzyme group protects the germ cell from free radical damage (Voganathan *et al.*, 1989*a*) and the effects of carcinogens and mutagens.

In this study GST isoenzyme expression was examined by immunohistochemistry, in normal and cryptorchid testes, ITGCN, seminoma and non-seminomatous germ cell tumours. We include a group of teratoma cases in which pre- and post-chemotherapy tissues were available.

### Materials and methods

### *Cases*

A total of 62 cases were studied. These were formalin-fixed, paraffin embedded biopsies from archives (1977–1990) where clinical follow-up data were available. These included 5 normal testes, seven cryptorchid testes and 11 cases of ITGCN. Thirty-nine cases of invasive germ cell tumours were studied composed of 22 malignant teratoma intermediate (MTI), eight malignant teratoma undifferentiated (MTU), six seminoma and three yolk sac tumours (British classification) (Pugh & Cameron, 1976, pp. 202–204). The age range of the tumour cases at time of diagnosis was 15–51 years. The normal testes were of histologically normal cases in males of 50 years and over, who had not received any hormonal therapy. In 16 stage II–IV MTI (Royal Marsden Hospital stage) (Heydig *et al.*, 1980) both orchidectomy specimens and post-treatment residual surgical masses were available for study (Table I).

### *Immunostaining*

Polyclonal antisera raised in the rabbit to GST Pi, Mu, Alpha and microsomal classes were the kind gift of Dr J.D. Hayes, University Department of Clinical Biochemistry, Edinburgh. Isoenzyme preparation, rabbit immunisation and characterisation of antibodies have been previously described (Morgenstern *et al.*, 1990; Hayes *et al.*, 1983; Hayes *et al.*, 1987; Hayes *et al.*, 1986).

Sections of formalin-fixed, paraffin-embedded tissues were cut at 3 μm, dewaxed in xylene and incubated with primary antibody at a dilution of 1:200 in phosphate buffered saline for a period of one hour at 20°C. Detection was by biotinylated swine anti-rabbit immunoglobulin (Dako) and streptavidin-biotin-peroxidase complex (Dako). Visualisation

Correspondence: H.S. Klys, Department of Pathology, University Medical School, Teviot Place, Edinburgh, UK.
Received 10 March 1992; and in revised form 14 May 1992.

**Table I** Treatment regimes and survival data for 16 stage II–IV malignant teratoma intermediate.

| Case | Stage at presentation | Treatment | Status | Duration of follow-up (months) |
|---|---|---|---|---|
| 1 | IVL1 | BEP × 4 | DIED | 8 |
| 2 | II B | BPV/E × 4 | A + W | 72 |
| 3 | II B | BEP × 4 | A + W | 48 |
| 4 | II C | BVP/E × 4 | A + W | 66 |
| 5 | II C | BPV/E × 4 | A + W | 62 |
| 6 | II A | BEP × 4 | A + W | 62 |
| 7 | IV H + | PVB × 5 | DIED | 25 |
| 8 | II A | PVB × 6 | DIED | 17 |
| 9 | II A | PVB × 5 | DIED | 10 |
| 10 | II B | BPV/E × 6 | A + W | 36 |
| 11 | IV CL2 | PVBEM × 6 | A + W | 60 |
| 12 | II C | BEP × 6 | A + W | 13 |
| 13 | III A | BPV/E × 4 | A + W | 59 |
| 14 | IV CL2 | BEP × 5 | A + W | 55 |
| 15 | III C | PBVEM × 6 | A + W | 55 |
| 16 | II C | BEP × 4 | A + W | 94 |

B = Bleomycin, E = Etoposide, V = Vinblastine, P = Cisplatin, M = Methotrexate, A + W = Alive and well.

using 3,3,-diaminobenzidine as substrate produced a brown insoluble precipitate. Sections were lightly counterstained with haematoxylin. Liver tissue was used as a control, GST Pi staining bile duct epithelium and GST Alpha, Mu and microsomal classes staining hepatocytes.

### Results

*Normal and cryptorchid testes*

Sertoli cells and Leydig cells showed strong positive cytoplasmic staining for GST Pi, Alpha, Mu and microsomal classes in all 5 normal testes. GST Pi was also present in nuclei (Figure 1). Spermatogonia and primary spermatocytes were negative in all normal testes. Faint reactivity of secondary spermatocytes and spermatids for GST Pi and Mu classes was seen in each case. In seven testes where histologically normal epididymal tissues were present, the epididymal lining epithelium was strongly positive for both GST Pi and Alpha. Six cases were positive for GST Mu and four for GST microsomal class.

In the seven cryptorchid testes there was a similar pattern of Leydig and Sertoli cell GST expression to the normal testis but the intensity appeared stronger (Figure 2).

*ITGCN*

The neoplastic germ cells in 11 cases of ITGCN showed strong nuclear and peripheral cytoplasmic staining for GST Pi (Figure 3). Faint staining for GST Alpha was present in six cases. GST Mu positivity was present in five cases and GST microsomal in four cases.

**Figure 2** Intense staining for GST Pi isoenzyme in the Leydig cells and Sertoli cells of the cryptorchid testis. (bar = 500 μm)

*Seminoma*

GST Pi was strongly expressed in all eight cases (Figure 4) with occasional negative cells and showed a similar cellular distribution to ITGCN. GST Alpha was positively expressed in seven cases, GST Mu in five cases, and GST microsomal in four cases.

*Teratoma*

All well differentiated epithelial elements of the 6 stage I MTI and 16 stage II–IV MTI testes showed strong expression of GST Pi (Figure 5a) and Alpha classes (Figure 5b). Mesenchymal elements (cartilage and smooth muscle), where present, were uniformly negative for GST Alpha class but strongly positive for GST Pi class (Figure 5). Undifferentiated elements (embryonal carcinoma) showed strong diffuse predominantly nuclear positivity for GST Pi in all cases. GST Alpha was negative for undifferentiated elements in three of 22 cases of MTI. In 19 positive cases of MTI the undifferentiated elements showed a weak background positivity with strong focal staining for GST Alpha related to areas of tubular and papillary differentiation (Figure 6). GST Mu and microsomal were positive in all cases of MTI but did not show any consistent relation to epithelial or mesenchymal differentiation.

There was no significant difference between staining pat-

**Figure 1** GST Pi isoenzyme expression in the normal testis. There is strong staining of Leydig cells with weaker positivity in Sertoli cells. (arrows) (bar = 50 μm)

**Figure 3** GST Pi isoenzyme expression in the neoplastic germ cells of ITGCN. (bar = 50 μm)

**Figure 4** Expression of the GST Pi isoenzyme in seminoma. (bar = 50 μm)

**Figure 5** **a** Expression of GST Pi isoenzyme in MTI chondrocytes and well differentiated epithelium. (bar = 125 μm). **b** Expression of GST Alpha isoenzyme in MTI well differentiated epithelium. The chondrocytes are negative. (bar = 125 μm)

terns for GSTs Pi and Alpha in MTI Stage I and MTI Stage II–IV.

The pattern of expression in post-treatment metastatic deposits of MTI Stage II–IV was similar to the primary tumour and reflected the component elements (differentiated epithelium, mesenchyme, embryonal carcinoma). There were no significant differences in isoenzyme expression between survivors and those who died of their disease.

**Figure 6** Focal expression of GST Alpha isoenzyme in embryonal carcinoma (MTU) in relation to tubulo-papillary formations. (bar = 125 μm)

The eight cases of MTU showed strong diffuse mainly nuclear staining for GST Pi. GST Alpha expression was similar to the undifferentiated elements of MTI; with focal strong positivity associated with tubular formations. All eight cases expressed GST Mu and four cases were positive for GST microsomal class. Three cases of yolk sac tumour showed positive expression of GST Pi, Alpha, Mu and microsomal classes.

### Discussion

We have demonstrated that GST Pi, Alpha, Mu and microsomal expression is present in and largely confined to the Sertoli and Leydig cell compartments of the normal human testis. Normal spermatogenesis is critically dependent on the close association of active Sertoli cells and developing germ cells (Ritzen *et al.*, 1981). Sertoli cells are largely responsible for germ cell oxido-reductive enzyme systems (Voganathan *et al.*, 1989*a*) and glutathione production (Li *et al.*, 1989). Testes of mature rodents contain high concentrations of GSH (Mushawar & Koeppe, 1973) and increase in testicular GSH parallels spermatogenic cell development (Grosshans & Calvin, 1985). GSH protects against germ cell mutagenesis by ethyl methanesulfonate in the F-344 rat and this is dependent on enzymatic conjugation by GST (Teaf *et al.*, 1985). GSTs may be an important enzyme system in detoxification of xenobiotics in the human testis but the role of individual carcinogens and teratogens is largely unknown. The demonstration of GST expression in the epididymis parallels previous studies in the rat testis (Hales *et al.*, 1980). At this site they may be important in final stages of spermatozoal maturation and provide protection against teratogens. The function of GSTs in the Leydig cell has not been defined. They have a role in steroid transport and isomerisation (Listowsky *et al.*, 1988) and this may also be of importance in the Sertoli cell. GST Alpha is present in the corresponding cell in human ovary, the enzymatically active stromal cell (EASC) (Rahilly *et al.*, 1991). The nuclear distribution of GST Pi has been previously described but its significance remains uncertain (Rahilly *et al.*, 1991).

We demonstrated GST Pi expression in ITGCN but not in normal spermatogonia. This is consistent with its expression in preneoplasia in other organs (Sato, 1989) and it shows a striking parallel with expression of placental alkaline phosphatase (PLAP). This latter enzyme is expressed in fetal germ cells, ITGCN (Hustin *et al.*, 1987) and seminoma and is used as a marker in routine diagnosis. GST Pi appears to reliably distinguish ITGCN from normal spermatogonia but is not such a specific marker. It has been shown that cisplatin-based chemotherapy may not eradicate ITGCN in some cases (Fossa & Aass, 1989; Chong *et al.*, 1986) implying a drugresistant phenotype and/or protection by the blood-testis barrier; and this may be important in development of a second primary tumour in the contralateral testis.

In seminoma and teratoma GST Pi was expressed in all tumour elements, irrespective of degree or line of differentiation. We were unable to demonstrate any qualitative difference between untreated primary tumours and post-chemotherapy metastatic deposits nor correlate expression with survival. However, the immunohistochemical method is relatively insensitive to changes in levels of expression and the study was necessarily limited by the availability of tissue from patients for whom follow-up data was available. GST Pi expression has been correlated with cisplatin resistance in human tumour cell lines, (Nakagawa *et al.*, 1988; Teicher *et al.*, 1987) but the implications in the clinical situation are uncertain. Raised cellular GSH levels protect against Bleomycin cytotoxicity (Russo *et al.*, 1984) but there is no direct evidence of GSH/GST involvement in resistance to vinca alkaloids or etoposide (VP-16).

We have shown that GST Alpha class expression is related to epithelial differentiation and that in embryonal carcinoma (MTU) expression is focal and related to tubal and papillary formations. In post-chemotherapy surgically resected masses differentiated elements are often the only viable component. It is believed that this is due to selective destruction of more primitive elements rather than chemotherapy-induced differentiation (McCartney *et al.*, 1984); and implies that differentiated elements are drug resistant, even though they behave in a less malignant fashion.

In summary GSTs are widely expressed in normal and neoplastic testicular tissues. They may be important in spermatogenesis and resistance to teratogens and carcinogens in the normal testis. They show characteristic patterns of expression in testicular tumours but their role in drug resistance is not well defined. GST Pi is a new marker for ITGCN.

### References

BATATA, M.A., CHU, F.C.H., HILARIS, B.S., WHITMORE, W.F. & GOLBEY, R.B. (1982). Testicular cancer in cryptorchids. *Cancer*, **49**, 1023–1030.

BATIST, G., TULPULE, A., SINHA, B.K., KATKI, A.G., MYERS, C.E. & COWANS, K.H. (1986). Overexpression of a novel anionic glutathione transferase in multi-drug-resistant human breast cancer cells. *J. Biol. Chem.*, **261**, 15544–15549.

CHASSEAUD, L.F. (1979). The role of glutathione and glutathione S-transferase in the metabolism of chemical carcinogens and other electrophilic species. *Adv. Cancer Res.*, **29**, 175–274.

CHONG, C., LOGOTHESIS, C.J., VON ESCHENBACH, A., AYALA, A. & SAMUELS, M. (1986). Orchiectomy in advanced germ cell cancer following intensive chemotherapy: comparison of systemic to testicular response. *J. Urol.*, **136**, 1221–1223.

DECLON, P.C. & BORCH, R.F. (1987). Characterisation of the reactions of platinum antitumour agents with biologic and non-biologic sulphur containing nucleophiles. *Biochem. Pharmacol.*, **36**, 1955–1964.

EINHORN, L.H. & WILLIAMS, S.D. (1980). Chemotherapy of disseminated testicular cancer. *Cancer*, **46**, 1339–1344.

FOSSA, S.D. & AASS, N. (1989). Cisplatin-based chemotherapy does not eliminate the risk of a second testicular cancer. *Br. J. Urol.*, **63**, 531–534.

GONDOS, B. & MIGLIOZZI, J.A. (1987). Intratubal germ cell neoplasia. *Semin. Diagn. Pathol.*, **4**, 292–303.

GROSSHANS, K. & CALVIN, H.I. (1985). Estimation of glutathione in purified population of mouse testis germ cells. *Biol. Reprod.*, **33**, 1197–1205.

HALES, B.F., HACHEY, C. & ROBAIRE, B. (1980). The presence of longitudinal distribution of GSH S-transferase in rat epithelium and vas deferens. *Biochem. J.*, **189**, 135–140.

HAYES, B.F., GILLIGAN, D., CHAPMAN, B.J. & BECKETT, G.J. (1983). Purification of human hepatic glutathione S-transferases and the development of radioimmunoassay for their measurement in plasma. *Clin. Chim. Acta.*, **134**, 107–121.

HAYES, J.D. & MANTLE, T.J. (1986). Use of immunoblot techniques to discriminate between the glutathione S-transferase Yf, Yk, Ya, Yn/Yb and Yc subunits and to study their distribution in extra-hepatic tissues. *Biochem. J.*, **233**, 779–788.

HAYES, J.D., MCLELLAN, L.I., STOCKMAN, P.K., CHALMERS, J. & BECKETT, G.J. (1987). Glutathione S-transferase in man: the relationship between rat and human enzymes. *Biochem. Soc. Trans.*, **15**, 721–725.

HEYDIG, W.F., BARRETT, A., MCELWAIN, T.J., WALLACE, D.M. & PECKHAM, M.J. (1980). The role of surgery in the combined management of metastases from malignant teratomas of the testis. *Br. J. Urol.*, **52**, 39–44.

HOWIE, A.F., FORRESTER, L.M., GLANCEY, M.J., SCHLAGER, J.J., POWIS, G., BECKETT, G.J., HAYES, J.D. & WOLF, C.R. (1990). Glutathione S-transferase and glutatione peroxidase expression in normal and tumour human tissues. *Carcinogenesis*, **11**, 451–458.

HUSTIN, J., COLLETTE, J. & FRANCHIMONT, P. (1987). Immunohistochemical demonstration of placental alkaline phosphatase in various stages of testicular development and in germ cell tumours. *Int. J. Androl.*, **10**, 29–35.

KRAMER, R.A., ZAKHER, J. & KIM, G. (1988). Role of the glutathione redox cycle in acquired and de novo multidrug resistance. *Science*, **241**, 694–697.

LAWRENCE, R.A. & BURK, R.F. (1978). Species, tissues and subcellular distribution of non Se-dependent glutathione peroxidase activity. *J. Nutr.*, **108**, 211–215.

LI, L., SEDDON, A.P., MEISTER, A. & RISLEY, M.G. (1989). Spermatogenic cell-somatic cell interactions are required for maintenance of spermatogenic cell Glutathione. *Biol. Reprod.*, **40**, 317–331.

LISTOWSKY, I., ABROMOVITZ, M., HOMMA, H. & NIITZU, V. (1988). Intracellular binding and transport of hormones and xenobiotics by glutathione S-transferase. *Drug Met. Rev.*, **19**, 305–318.

MCCARTNEY, A.C.E., PARADINAS, F.J. & NEWLANDS, E.S. (1984). Significance of the 'maturation' of metastases from germ cells after intensive chemotherapy. *Histopathology*, **8**, 457–467.

MANNERVIK, B. (1985). The isoenzymes of Glutathione transferase. *Adv. Enzymol.*, **57**, 357–417.

MANNERVIK, B., ALIN, P., GUTHENBERG, C., JENSSON, H., TAHIR, M.K., WARHOLM, M. & JORNVALL, H. (1985). Identification of three classes of cytosolic glutathione transferase common to several mammalian species: correlation between structural data and enzymatic properties. *Proc. Natl Acad. Sci. USA.*, **82**, 7202–7206.

MANNERVIK, B. & DANIELSON, V.H. (1988). Glutathione transferases-structure and catalytic activity. *Crit. Rev. Biochem.*, **23**, 283–337.

MORGENSTERN, R., LUNDQVIST, G., MOSIALOU, E. & ANDERSSON, C. (1990). Membrane bound glutatione transferase: function and properties. In *Glutathione S-Transferase and Drug Resistance*. Taylor and Francis: London. pp. 57–64.

MUSHAWAR, I.K. & KOEPPE, R.E. (1973). Free amino acids of testes. Concentrations of free amino acids in the testes of several species and the precursors of glutamate and glutamine in rat testes *in vivo*. *Biochem. J.*, **254**, 5184–5190.

NAKAGAWA, K., YOKOTA, J. & WADA, M. (1988). Levels of glutathione S-transferase pi mRNA in human lung cancer cell lines correlate with resistance to cisplatin and carboplatin. *Jpn. J. Cancer Res.*, **79**, 301–304.

PUGH, R.C.B. & CAMERON, K.M. (1976). In *Pathology of the Testis*. Blackwell: Oxford. pp 202–204.

RAHILLY, M.A., CARDER, P.J., AL-NAFUSSI, A. & HARRISON, D.J. (1991). Distribution of glutathione S-transferase isoenzymes in normal ovary. *J. Reprod. Fertil.*, **93**, 303–311.

RITZEN, E.M., HANSSON, V. & FRENCH, F. (1981). *The Testis*. New York: Raven Press, 171–205.

RUSSO, A., MITCHELL, J.B., MCPHERSON, S. & FRIEDMAN, N. (1984). Alteration of bleomycin cytotoxicity by glutathione depletion or elevation. *Int. J. Radiat. Oncol. Biol. Phys.*, **10**, 1675–1678.

SATO, K., SATOH, K. & HATAMAYO, I. (1987). Placental Glutathione S-transferase as a marker for preneoplastic tissues. Glutathione S-transferases and carcinogenesis pp 127–138. Taylor and Francis.

SATO, K. (1989). Glutathione transferase as markers of preneoplasia and neoplasia. *Adv. Cancer Res.*, **52**, 205–255.

TEAF, C.M., HARBISON, R.D. & BISHOP, J.B. (1985). Germ-cell mutagenesis and GSH depression in reproductive tissue of the F-344 rat induced by ethyl methanesulfonate. *Mutat. Res.*, **144**, 93–98.

TEICHER, B.A., HOLDEN, S.A., KELLY, M.J., SHEA, T.C., CUCCHI, C.A., ROSOWSKY, A., HENNER, W.D. & FREI, E. (1987). Characterisation of a human squamous carcinoma cell line resistant to cisdiammine dichloroplatinum. *Cancer Res.*, **47**, 388–393.

VOGANATHAN, T., ESKILD, W. & HANSSON, V. (1989*a*). Investigation of detoxification capacity of rat testicular germ cells and Sertoli cells. *Free Rad. Biol. Med.*, **7**, 355–359.

VOGANATHAN, T., OYEN, O., ESKILD, W. & JAHNSEN, T. (1989*b*). Cellular localisation and age-dependent changes in m RNA for Glutathione S-transferase–P in rat testicular cells. *Biochem. Int.*, **19**, 667–672.

WAXMAN, D.J. (1990). Glutathione S-transferases: Role in alkylating agent resistance and possible target for modulation chemotherapy–A Review. *Cancer Res.*, **50**, 6449–6454.